# Supplementary material for: The Association between Ankle–Brachial Index/Pulse Wave Velocity and Cerebral Large and Small Vessel Diseases in Stroke Patients
Source: Diagnostics (Basel). 2023 Apr 18;13(8):1455. doi: 10.3390/diagnostics13081455 (PMC10137399; doi:10.3390/diagnostics13081455)
Supplement: Supplementary file 1 [file diagnostics-13-01455-s001.zip › diagnostics-2259888-supplementary.pdf]

Figure S1: The study flowchart

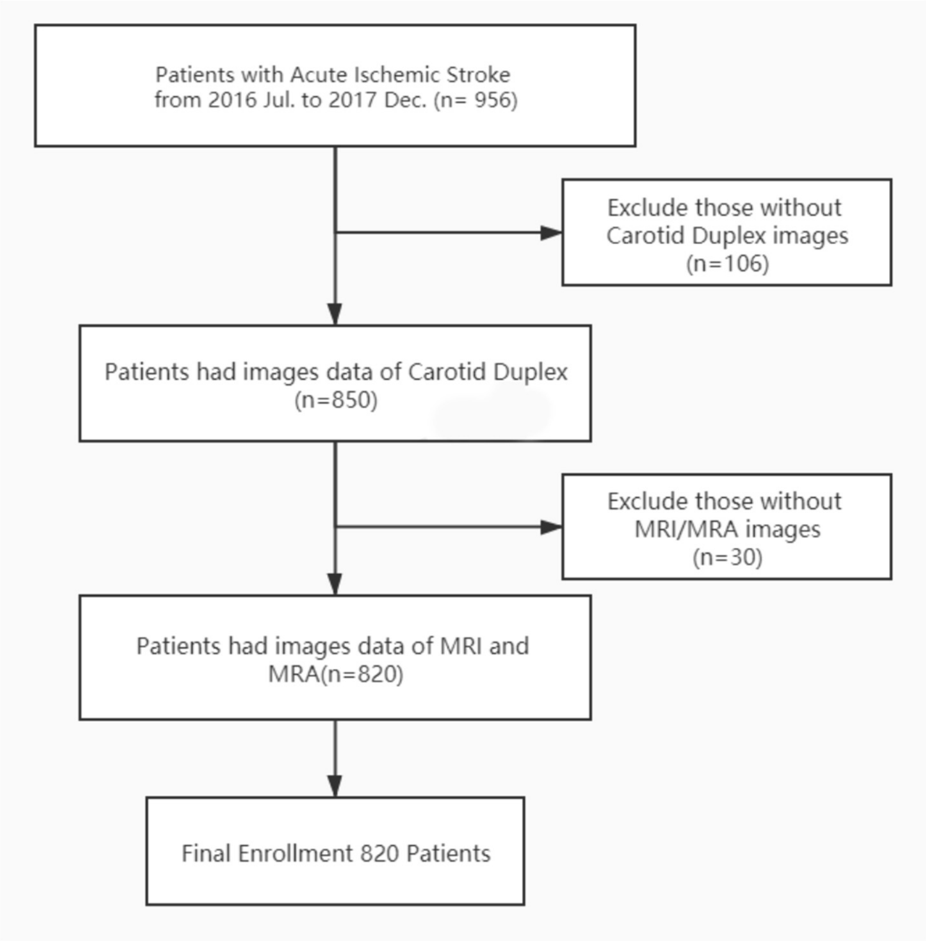

Table S1. Demographic data of enrolled participants

	N	n (%) / Median (IQR)
**Characteristic**		
Age	820	68.00 (59.00, 78.00)
Male	820	501 (61.10)
Body height	819*	162.00 (155.00, 168.00)
Body Weight	819*	62.00 (55.00, 71.00)
BMI	819*	24.03 (21.49, 26.64)
Af	641*	91 (14.20)
HTN	820	535 (65.24)
DM	820	319 (38.90)
Hyperlipidemia	820	422 (51.46)
Heart disease	820	112 (13.66)
Dementia	795*	33 (4.15)
**Measurement of arterial indicators**		
ABI, average	820	1.08 (1.01, 1.14)
ABI, minimum	820	1.06 (0.97, 1.11)
BaPWV, average	785*	18.90 (16.10, 21.80)
BaPWV, maximum	785*	19.50 (16.70, 22.90)
**Cerebral neuroimaging markers**		
Any Extracranial stenosis	820	743 (90.61)
Total Extracranial stenosis grade	820	5.00 (4.00, 6.00)
Any Intracranial stenosis	820	551 (67.20)

Total Intracranial stenosis grade	820	11.00 (9.00, 14.00)
Any Presence of SVD	820	492 (60.00)
Total SVD scores	820	
0		328 (40.00)
1		157 (19.15)
2		169 (20.61)
3		100 (12.20)
4		66 (8.05)

*Some patient's data was missing from the medical records or not to be checked during hospitalization

ABI: Ankle-brachial index; BMI: body mass index; baPWV: brachial-ankle pulse wave velocity; HTN: hypertension; DM: diabetes mellitus; Af: atrial fibrillation; SVD: small vessel disease

* Average ABI/baPWV indicated the average of bilateral ABI/baPWV values; minimum ABI indicated the lowest data of ABI measured on bilateral sides; maximal baPWV indicated the highest data of baPWV measured on both sides.

Table S2. ABI and CSVD score sub-items

	N	ABI, average Median (IQR)	P value [a]	ABI, minimum Median (IQR)	P value [a]
Lacunes					
Absence (Score 0)	476	1.09 (1.03, 1.14)	<0.001	1.07 (1.00, 1.12)	<0.001
Presence(Score 1)	344	1.07 (0.98, 1.12)		1.04 (0.93, 1.10)	
Microbleeds					
Absence (Score 0)	585	1.08 (1.02, 1.14)	0.233	1.06 (0.98, 1.11)	0.158
Presence(Score 1)	235	1.08 (0.99, 1.13)		1.05 (0.96, 1.11)	
Perivascular spaces					
Absence to Mild (Score 0)	537	1.09 (1.03, 1.14)	<0.001	1.07 (1.00, 1.12)	<0.001
Moderate to Severe(Score 1)	283	1.06 (0.98, 1.13)		1.03 (0.92, 1.10)	
White matter intensities					
Absence to Mild (Score 0)	623	1.09 (1.03, 1.14)	<0.001	1.07 (0.99, 1.12)	<0.001
Moderate to Severe(Score 1)	197	1.05 (0.94, 1.12)		1.02 (0.88, 1.08)	

[a] Mann-Whitney U test.

* Average ABI indicated the average of bilateral ABI values; minimum ABI indicated the lowest data of ABI measured on bilateral sides.

ABI: Ankle-brachial index

Table S3. BaPWV and CSVD score sub-items

	N	BaPWV, average Median (IQR)	P value [a]	BaPWV, maximum Median (IQR)	P value [a]
Lacunes					
Absence (Score 0)	456	18.30 (15.55, 21.30)	<0.001	18.80 (16.20, 22.05)	<0.001
Presence(Score 1)	329	19.70 (17.10, 22.60)		20.40 (17.80, 23.60)	
Microbleeds					
Absence (Score 0)	566	18.70 (15.95, 21.60)	0.030	19.20 (16.50, 22.50)	0.006
Presence(Score 1)	219	19.40 (16.65, 22.30)		20.20 (17.40, 23.50)	
Perivascular spaces					
Absence to Mild (Score 0)	521	18.30 (15.65, 21.30)	<0.001	18.90 (16.30, 22.10)	<0.001
Moderate to Severe(Score 1)	264	20.00 (17.38, 22.98)		20.70 (18.00, 24.00)	
White matter intensities					
Absence to Mild (Score 0)	602	18.40 (15.80, 21.35)	<0.001	19.00 (16.40, 22.20)	<0.001
Moderate to Severe(Score 1)	183	20.80 (17.70, 23.00)		21.40 (18.30, 24.30)	

[a] Mann-Whitney U test.

* Average baPWV indicated the average of bilateral baPWV values; maximum baPWV indicated the highest data of baPWV measured on bilateral sides.

BaPWV: brachial-ankle pulse wave velocity
